# Expression of transmembrane carbonic anhydrases, CAIX and CAXII, in human development

**DOI:** 10.1186/1471-213X-9-22

**Published:** 2009-03-16

**Authors:** Shu-Yuan Liao, Michael I Lerman, Eric J Stanbridge

**Affiliations:** 1 Department of Pathology, St. Joseph Hospital, Orange, CA, USA; 2 Department of Epidemiology, School of Medicine, University of California at Irvine, Irvine, CA. 94697, USA; 3 Center for Cancer Research, National Cancer Institute-Frederick, Frederick, MD. 21702, USA; 4 Department of Microbiology & Molecular Genetics, School of Medicine, University of California at Irvine, Irvine, CA. 94697, USA

## Abstract

**Background:**

Transmembrane CAIX and CAXII are members of the alpha carbonic anhydrase (CA) family. They play a crucial role in differentiation, proliferation, and pH regulation. Expression of CAIX and CAXII proteins in tumor tissues is primarily induced by hypoxia and this is particularly true for CAIX, which is regulated by the transcription factor, hypoxia inducible factor-1 (HIF-1). Their distributions in normal adult human tissues are restricted to highly specialized cells that are not always hypoxic. The human fetus exists in a relatively hypoxic environment. We examined expression of CAIX, CAXII and HIF-1α in the developing human fetus and postnatal tissues to determine whether expression of CAIX and CAXII is exclusively regulated by HIF-1.

**Results:**

The co-localization of CAIX and HIF-1α was limited to certain cell types in embryonic and early fetal tissues. Those cells comprised the primitive mesenchyma or involved chondrogenesis and skin development. Transient CAIX expression was limited to immature tissues of mesodermal origin and the skin and ependymal cells. The only tissues that persistently expressed CAIX protein were coelomic epithelium (mesothelium) and its remnants, the epithelium of the stomach and biliary tree, glands and crypt cells of duodenum and small intestine, and the cells located at those sites previously identified as harboring adult stem cells in, for example, the skin and large intestine. In many instances co-localization of CAIX and HIF-1α was not evident. CAXII expression is restricted to cells involved in secretion and water absorption such as parietal cells of the stomach, acinar cells of the salivary glands and pancreas, epithelium of the large intestine, and renal tubules. Co-localization of CAXII with CAIX or HIF-1α was not observed.

**Conclusion:**

The study has showed that: 1) HIF-1α and CAIX expression co- localized in many, but not all, of the embryonic and early fetal tissues; 2) There is no evidence of co-localization of CAIX and CAXII; 3) CAIX and CAXII expression is closely related to cell origin and secretory activity involving proton transport, respectively. The intriguing finding of rare CAIX-expressing cells in those sites corresponding to stem cell niches requires further investigation.

## Background

Carbonic anhydrases are metalloenzymes that are ubiquitous in nature, being found in prokaryotes and eukaryotes [1-3]. They are encoded by five distinct, evolutionarily related gene families (α, β, γ, δ and ζ). The complexity of the family membership increases with evolutionary progress and, in mammals, which contain 16 α-CA isozymes, the distribution of expression of family members differs enormously, varying from expression in virtually all cells (CAII) to very discrete temporal and tissue specific expression (CAIX, CAXII and CAXIV). Furthermore, the cellular localization of the CAs also varies, including cytoplasmic, mitochondrial, transmembrane and secreted [2-9].

The transmembrane CAIX and, to a lesser degree, transmembrane CAXII, have received a great deal of attention. This is because, in large measure, CAIX has been shown to be an excellent cellular biomarker of hypoxia [7,10,11] and a biomarker of certain malignancies, including renal clear cell carcinoma [12,13] and cervical dysplasia and carcinoma [14].

The association with hypoxia has been ascribed functionally to the fact that the CA9 promoter contains a hypoxia response element (HRE) that is the target for the hypoxia inducible factor 1 (HIF-1) transcription factor [10]. The alpha subunit of the heterodimeric HIF-1 (HIF-1α) is rapidly degraded under normoxic O2 levels but is stable under hypoxic conditions. Thus, HIF-1 is stable and transcriptionally active predominantly in hypoxic tissues. This close correlation between hypoxia and CAIX expression has led to studies that have identified CAIX expression in tumor cells that reside in hypoxic regions of solid tumor tissues, i.e. those most refractory to radiation treatment and certain drug modalities, as a potential therapeutic target [2,3,15].

Although it is clear that the CA9 gene is a transcriptional target of HIF-1 there have been several studies that have shown substantial but incomplete co-localization of CAIX expression and regions of hypoxia in solid tumors, with CAIX-positive areas extending beyond the region of hypoxia [7,10,11]. Other studies also showed that CA9 transcription is up-regulated by extracellular acidosis [7,16] and negatively controlled by an epigenetic mechanism that involves methylation of the CA9 promoter [17]. In addition to transcriptional regulation, control of CAIX expression may involve phosphatidyl inositol 3- kinase (P13-kinase) activity [18] or alternative splicing of the CA9 transcript [19].

Moreover, our previous studies also observed HIF-1α positive nuclear staining in squamous cells lining the oral cavity, esophagus and cervix but no CAIX expression was observed (data not shown). All of these studies would suggest the possibility that transcriptional factors, other than HIF-1, may modulate CAIX expression. Furthermore, although CA12 was initially thought to be a HIF-responsive gene [10], other evidence has questioned this correlation [7,20]. Our previous study of CAIX and CAXII expression in human tumor and normal adult tissues had also showed that co-expression of CAIX and CAXII in tumor tissue was mainly restricted to regions of hypoxia, but in normal adult tissues CAIX and CAXII expression did not appear to be induced only by hypoxia and seems to be related to the functional status and cell origin of the relevant tissue.

It has been well established that the mammalian embryo and early stages of fetal development are hypoxic [21-24]. As the process of vascularization occurs hypoxic tissues become progressively normoxic. Thus, the study of the distribution of CAIX, CAXII and HIF-1α expression in the developing human embryo and fetus would be an ideal in vivo system in which to determine whether the postulated coordination of hypoxia, HIF-1 activity and induction of expression of CAIX and CAXII is seen, and also to test our hypothesis that CAIX and CAXII expression may be related to cell origin and secretory activity involving proton transport.

## Results and Discussion

During embryonic development, cytotrophoblasts are derived from the trophoblasts of the blastocyst. Later the cytotrophoblasts, together with the coelomic membrane and extraembryonic somatic mesoderm, constitute the chorion. The extraembryonic somatic mesoderm forms the connecting stalk (later becoming the umbilical cord). During the 3rd week (gastrulation), many cells of the epiblast detach themselves from the neighboring cells and form the mesoblast. The cells that remain in the epiblast eventually form the embryonic ectoderm. Some of migrating mesoblastic cells organize and form the intraembryonic mesoderm and the notochord process. Some mesoblastic cells form a loosely woven tissue of the embryonic mesenchyme. These mesenchymal cells have the potential to proliferate and differentiate into diverse types of cells (fibroblasts, chondroblasts, osteoblasts, myoblasts, etc.). At the end of the 3rd week, the intraembryonic coelom develops from the lateral and cardiogenic mesoderm and eventually gives rise to the body cavity [25]. It is at this stage that we were able to obtain our earliest embryonic tissue for analysis.

### CAIX expression during the embryonic period (4 to 8 weeks)

**Figure 1:**
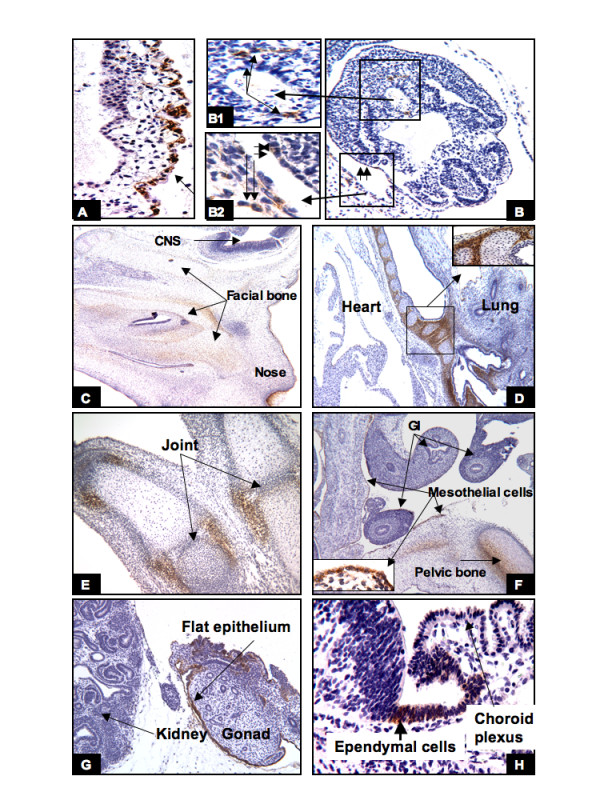
**The embryonic period (4–8 weeks)**: CAIX expression was first seen in the cytotrophoblasts (A, arrow) and certain cells in the embryo (B), such as primitive mesenchyma (B1 long arrow), external surface of the embryo (B2, short double arrows), and mesenchymal cells of the chorion (B2, double long arrows). In the later embryonic stage, CAIX expression was primarily observed in the mesencyhmal cells involving chondrogenesis, as shown in the facial bone (C), bronchial tree (D), the limb (E), and the pelvic bone (F). All of the epithelial cells lining the body cavity were also positive for CAIX, e.g. the peritoneum (F), and the surface of the gonad (G). The only CAIX-positive cells in the CNS are ependymal cells (H). Original magnifications: A, B and H (20×); B1 and B2 (40×); C, D and F (4×); E and G (10×).

As early as at the 3rd to 4th week of gestation, CAIX expression was already present in the cytotrophoblasts (Fig. 1A, arrow) and rare mesenchymal cells of the chorion and the connecting stalk. In the embryo there were rare cells along the external surface (ectoderm) (Fig. 1B and 1B2, short arrows) and in the primitive mesenchyme (mesoblastic cells) that also weakly expressed CAIX (Fig. 1B1 and 1B2, long arrows). As gestation progresses, increased levels of CAIX expression were seen in the basal layer of the skin, and in the undifferentiated mesenchymal cells of the embryo involved in chondrogenesis. At 7 to 8 weeks, high levels of CAIX expression were seen in the perichondrial undifferentiated mesenchymal cells of the skeletal system, such as facial bone (Fig. 1C), the tracheobronchial cartilage (Fig. 1D), limbs and pelvic bone (Fig. 1E, 1F), and vertebrae (data not shown). All epithelial cells (mesothelial cells) and underlying mesenchyma lining the body cavities showed varying degrees of CAIX expression (Fig. 1F, 1G). As mentioned above, at the 3rd to 4th week of gestation there were only a relatively few weakly positive CAIX expressing cells identified in the embryo, and some of these cells appeared to locate near the neural plate. At 7 to 8 weeks of gestation, the only cells in the nervous system that expressed CAIX were the primitive ependymal cells, derived from the neural crest cells (Fig. 1H). It is worth noting that no other embryonic cells, besides the cells described above, expressed CAIX. For example, CAIX expression was not seen in the lung, heart (Fig. 1D), intestine (Fig. 1F), kidney or gonad (Fig. 1G).

### CAIX expression during fetal development (9 to 40 weeks)

During the fetal stage, CAIX expression was closely related to the cell origin (coelomic derivatives), state of cell differentiation, microenvironmental pH status and, to a large degree, the condition of hypoxia. Those tissues expressing CAIX during the embryonic period continued to retain high levels of CAIX expression during the first to 2nd trimester. However, with a few exceptions (body cavity lining cells, coelomic derived remnants, and the gastrointestinal tract), the levels of CAIX expression progressively diminished after 29–30 weeks of gestation and eventually vanished during the postnatal period. After the age of one year, CAIX expression is similar to that observed in normal adult tissues [7]. The details are as follows:

#### Placenta

**Figure 2:**
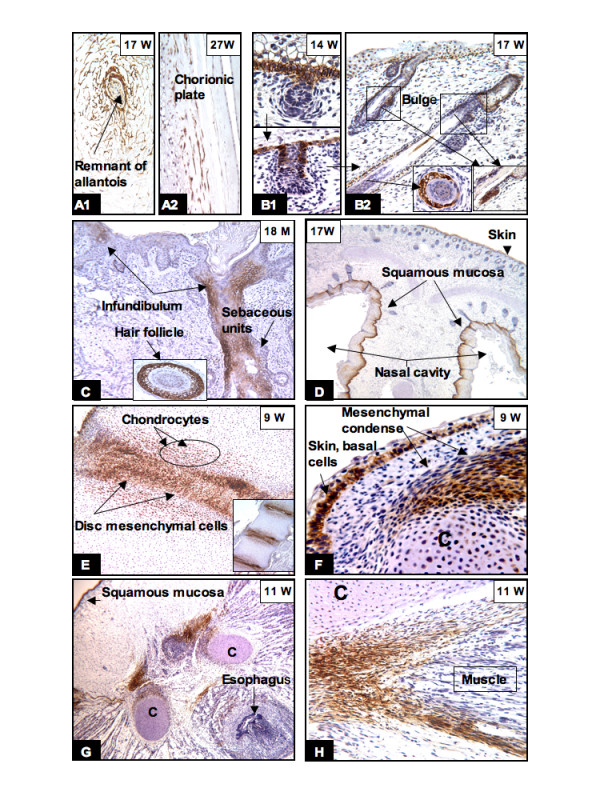
**CAIX expression in the placenta, skin, squamous mucosa and skeletal system**. The mesenchymal cells of the chorionic plate and umbilical cord retained their CAIX expression until birth (A1, A2). In the epidermis, CAIX expression was limited to the basal layer (B1, F), hair buds (B1) and sebaceous units, hair follicles and the bulges (B2, arrow and inserts). Between 18 to 24 months after birth, CAIX expression was restricted to the hair follicles, sebaceous units, and the infundibulum (C). In the skeletal system, persistent high levels of CAIX immunoreactivity was seen in the chondrocytes, mesenchymal condense (E, F, G) and tendoligamental tissues (G, H). The basal layer of squamous mucosa of the nose (D) and oral cavity (G) also expressed CAIX. W = gestational age in weeks; M = postnatal age in months. Original magnifications: A1, A2, B, C and H (20×); D and G (4×); E (10×) and F (40×).

High levels of CAIX expression in the mesenchymal cells of the connecting stalk/umbilical cord (Fig. 2A1) and the chorionic plate (Fig. 2A2) were persistent throughout fetal development to term pregnancy. No CAIX immunoreactivity was observed in the chorionic villi.

#### The body cavity

**Figure 3:**
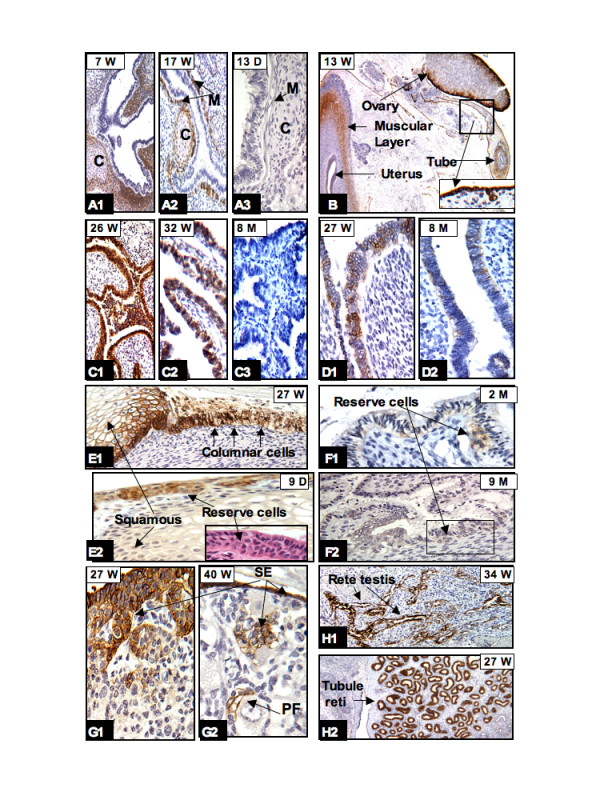
**CAIX expression in bronchial trees and genital organs:** In the bronchial trees high expression of CAIX was seen in the peribronchial immature mesenchymal cells and cartilage (designated C) (A1) but progressively diminished when the tissues became mature (A2, A3). CAIX positivity was persistently seen in peritoneal lining cells as shown in B (insert) and G1, G2 (surface epithelium [SE], arrow). As early as the 13th week of gestation, CAIX positive cells were seen in the flat SE of the ovary, the outer muscular layer and rare epithelial cells of the uterus and fallopian tubes (B). Around 26 to 27 weeks, high levels of CAIX expression were transiently seen in the epithelium of fallopian tubes (C1), endometrium (D1), and the cervix (E1). After birth, there was either no CAIX expression (C3) or expression was limited to occasional endometrial cells (D2), reserve cells of the cervix (E2, and corresponding H&E stain in insert, and F1, F2). In the ovary, CAIX expression was observed in SE migrating into the stroma and forming the primordial follicle (PF) (G1, G2). Persistent expression of CAIX was observed in the coelomic remnants: rete testis (H1) and tubule reti (H2). W = gestational age in weeks; D, M = postnatal age in days and months, respectively. Original magnifications: A1, A2, A3, C1, C2, C3, E1, E2 and F2 (20×); B (4×); H1 and H2 (10×); D1, D2, F1, G1 and G2 (40×).

The mesothelial cells lining the body cavities and the surfaces of visceral organ system showed high levels of CAIX expression throughout the developmental stages of the fetus and continued into adulthood [7]. Representative examples are shown in figs 1F, 1G, 3B, 3G1, and 3G2.

#### The skin and squamous mucosa

During the fetal period the basal cells of the skin continuously proliferate and eventually form the hair follicle, and the sebaceous, apocrine and eccrine units. In the early fetal stage, increasing CAIX expression was seen along the basal layer and the hair bud; however, no expression of CAIX was seen in the hair bulb and condensation of mesenchyma (Fig. 2B1). Around 16 to 17 weeks of gestation, high levels of CAIX expression were seen in the basal cells, sebaceous unit, the bulge of the prospective site of attachment of the erector muscle, and the outer sheath of the hair follicle (Fig. 2B2). The expression was persistent throughout the fetal period, but progressively diminished during the postnatal period. 18 to 24 months after birth, CAIX immunoreactivity was similar to that seen in the adult skin and was concentrated in the outer sheath of hair follicles, the sebaceous unit and the infundibulum of the hair follicle (Fig. 2C). However, scattered CAIX positive cells were also present along the basal layer between the infundibulo-sebaceous-apocrine units (data not shown). Besides the skin, the dental lamina, enamel organ and the basal cells of the squamous mucosa of the nose, the oral cavity, esophagus and the genital organ system, including the cervix, vagina and anus, also showed variable degrees of CAIX expression, ranging from expression limited to a few basal cells (data not shown) to diffuse basal cell positivity, as shown in fig 2D (the nose at 17 weeks of gestation). After 26 weeks, the levels of CAIX expression progressively diminished. At one year of postnatal age, CAIX expression was restricted to a few basal cells. However, increased levels of CAIX expression in the reparative basal cells were noticed when there was tissue repair due to injury or inflammation (data not shown).

#### The articular, skeletal and muscular system

There was progressively increased intensity of CAIX expression in the mesenchymal cells involved in chondrogenesis. By the 9th to 11th week of gestation, high levels of CAIX expression were seen in the primitive mesenchyme and chondroblasts in most parts of the skeletal system, especially in the articular disc of the vertebrae (Fig. 2E), the condensation of the mesenchyme around the immature cartilage of the limbs/facial bone (Fig. 2F, 2G), the primitive mesenchymal cells of tendoligamentous units (Fig. 2H) and the tracheobronchial trees (Fig. 3A1). These areas have been shown by us (data shown later) and others [24] to be predominantly hypoxic; thus, CAIX expression in these areas is most likely due to HIF-1 mediated gene expression. In the muscular system, CAIX positivity was observed around the 10th week of gestation. Although the levels of CAIX expression in the muscular system varied among the visceral organs it was often observed in peritracheal primitive muscle cells (Fig. 3A2), the external layer of the muscular propria of the genitourinary tract (Fig. 3B) and the gastrointestinal system, the aorta, and focally in cardiac muscle (data not shown). All of these tissues are derived from the splanchnic mesenchyma. In addition, in the heart there were specialized mesenchymal cells (coelomic origin) at the atrioventricular junction that also expressed CAIX (data not shown). After 20 weeks of gestation, CAIX expression progressively diminished and eventually disappeared when these mesenchymal cells differentiated into mature cartilage, joint capsule, ligament, synovium, and muscular propria of all visceral organs. An illustrative example of the progressive loss of CAIX expression during cell maturation is shown in Fig. 3A. Diffuse CAIX expression was seen in the peritracheobronchial primitive mesenchyma (Fig. 3A1). In the relatively well developed lung (after 17 weeks), CAIX expression was limited to the immature peritracheobronchial muscle and perichondrial mesenchymal cells (Fig. 3A2). After birth to one year of age, CAIX expression was no longer present in the cartilage or muscle (Fig. 3A3).

#### The genital system

During fetal development, all of the genital organs derived from the coelom and paramesonephric ducts (Müllerian duct) showed variable degrees of CAIX expression. These levels of expression can basically be divided into three periods (13 to 24 weeks; 25 to 28 weeks; and 29 weeks to birth). As early as at the 13th week of gestation, CAIX expression was identified in the primitive muscular layer of the uterus and fallopian tubes (Fig. 3B), the flat surface epithelium (modified mesothelium) of all male and female genital organs (Fig. 3B insert), and in rare epithelial cells of the endometrium, cervix and fallopian tubes. By the end of the 2nd trimester (24 weeks), there was no longer any observable CAIX expression in the muscular propria. However, there were transient increased levels of CAIX expression in the epithelial cells of the fallopian tube (Fig. 3C1), endometrium (Fig. 3D1), cervix (Fig. 3E1) and vagina during 26 to 28 weeks of gestation. CAIX expression progressively diminished after 29 weeks but the levels of expression appear to vary from case to case. Usually near or after one year of age, CAIX expression was either extinguished, as seen in the fallopian tube (Fig. 3C3), or restricted to rare columnar cells of the endometrium (Fig. 3D2). In the cervix, high levels of CAIX expression were persistently observed in the reserve cells (Fig. 3E2) and, to a lesser degree, in columnar cells at 1–2 months postnatally. Between 2–12 months postnatally, CAIX expression was restricted to rare reserve cells (Fig. 3F1, 3F2). In contrast, throughout the fetal period CAIX was persistently expressed in the flat surface epithelium of all genital organs and the coelomic derived remnants, such as the rete ovarii, rete testis and tubuli reti, the hydatid of Morgagni, and the appendix of the testis. Interestingly, the efferent ductules that merged into the rete testis also showed diffuse immunoreactivity for CAIX.

Representative illustrations are shown in figs 3G1, 3G2 (ovarian surface and primordial follicles), and figs 3H1, 3H2 (the rete testis and tubule reti). With the exception of mature follicles of the ovary, these expression patterns are continuously seen throughout adult life [7]

#### The urinary system

The urinary organs consist of the kidneys, ureters, urinary bladder, prostate and urethra. The kidneys and ureters are derived from the metanephrons, whereas the epithelium of the bladder, prostate and urethra are derived from the endoderm. With the exception of efferent ductules as described above, CAIX expression was limited to a few prostate terminal glands and rare primitive reserve cells of the urothelial epithelium (data not shown).

#### The endocrine and hematopoietic system

**Figure 4:**
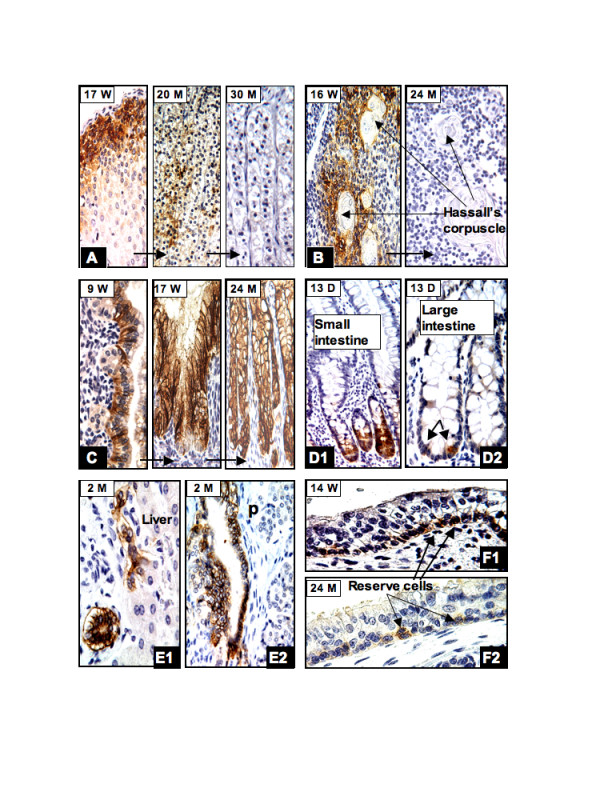
**In the adrenal gland (A), strong CAIX immunoreactivity was seen in many cortical cells near the capsule (17W), but the level of expression progressively diminished after birth and, by the end of two years, no CAIX positive cells were seen**. In the thymus gland (B), CAIX positive cells were primarily identified near the Hassall's corpuscles (16W) but disappeared by the first year after birth (24M). In the stomach (C), CAIX expression was first seen in surface columnar cells at the 9th week of gestation (9W). At 17 weeks, diffuse immunoreactivity was seen in the glandular and surface columnar cells (17W). This level of expression persisted after birth (24M). In the small intestine CAIX positive cells were restricted to the crypts (D1) and in the large intestine only rare positive cells were identified near the base of the crypts (D2). In the biliary trees, CAIX expression was seen in the epithelium of the entire ductal system and was persistent after birth. The liver (E1) and pancreas (E2) are shown as examples. During early fetal period, extensive CAIX expression was seen in the basal/reserve cells of the respiratory epithelium (F1, 14W) but after birth only rare reserve cells continued to express CAIX (F2, 24M). W = gestational age in weeks; D, M = postnatal age in days and months, respectively. Original magnifications: A1, A2, A3, B1 and C (20×); B2, D1, D2, E1, E2, F1 and F2 (40×).

None of the endocrine organs and hematopoietic systems expressed CAIX, with the exception of the adrenal cortical cells and the thymus gland. The proliferation of the coelomic epithelium initially forms the fetal cortex of the adrenal glands. Around 8 weeks of gestation, more coelomic epithelial cells proliferate and constitute the permanent cortex. CAIX immunostaining in the adrenal cortical cells was first seen around 12–14 weeks of gestation and reached its highest levels of expression during gestational age of 20–28 weeks. The CAIX immunoreactivity was persistent until birth but the numbers of positive cortical cells progressively decreased. By the end of 2 years, no CAIX positive cells were identified in the adrenal glands. Representative illustrations of this temporal sequence of expression are shown in fig 4A. The developing thymus gland comprises the mesenchyme, hematopoietic stem cells and the epithelial cell cords derived from the third pair of endodermal pharyngeal pouches. At the 13th week of gestation, scattered CAIX positive cells were identified near the Hassall's corpuscles. After the 17th week of gestation, high levels of CAIX expression extended to the surface of the lobules (Fig. 4B, 16 weeks). The cells that expressed CAIX did not appear to co-express cytokeratin (data not shown). From this we speculate that these CAIX positive cells were probably immature mesenchymal cells rather than thymic epithelial cells. As the thymus progressively matured the numbers of CAIX positive cells decreased and, after the age of one year, no CAIX expression was seen in the thymus (Fig. 4B, 24 Months).

#### The epithelial cells of the respiratory and gastrointestinal systems (endodermal derivatives)

CAIX expression in the GI system was persistent throughout fetal development. CAIX immunoreactivity was first observed in surface columnar cells of the stomach (Fig. 4C), and the crypt cells of the duodenum at 9–12 weeks of gestation. After 16–17 weeks CAIX expression was restricted to certain epithelial cells along the GI system, including gastric glandular cells, neck mucous cells and fundic glandular cells (Fig. 4C), the pyloric and Brunner's glands; the crypt cells of the duodenum and small intestine (Fig. 4D1); and the appendix. The numbers of CAIX immunostained crypt cells were significantly decreased in the distal part of the small intestine. CAIX expression in the large intestine was limited to rare cells in the crypts and was not clearly identified until late in the third trimester. After birth, the numbers of positive cells in the large intestine varied from case to case, but were consistently located near the bottom of the crypts (Fig. 4D2). High levels of CAIX expression were seen in the ductal cells of the entire biliary system, including bile ducts of the liver (Fig. 4E1), common bile ducts, gallbladder, and pancreatic ducts (Fig. 4E2). In the esophagus and the respiratory system, CAIX expression was seen diffusely distributed in the basal/reserve cells of the squamous and respiratory mucosa during the early fetal period (Fig. 4F1) but, after birth, the expression progressively decreased. At the age of one year and thereafter, CAIX immunoreactivity was limited to a few basal cells of the squamous mucosa and primitive reserve cells of the respiratory epithelium (Fig. 4F2).

#### The nervous system

During fetal development the only cells in the nervous system that expressed CAIX were the immature ependymal cells and leptomeningeal loose connective tissues of mesoderm derivatives. Its expression was persistent throughout the fetal period.

### CAIX expression during the postnatal period (after birth to 8 years old)

**Figure 5:**
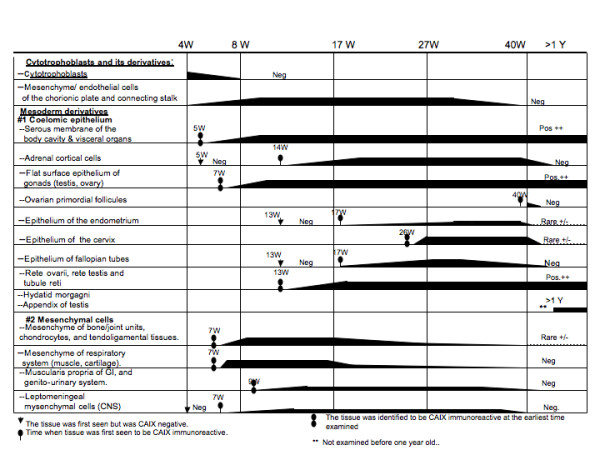
**Schema of CAIX expression in the cytotrophoblast and extra-/intra-embryonic mesoderm derivatives during human development (embryonic, fetal and postnatal periods)**.

**Figure 6:**
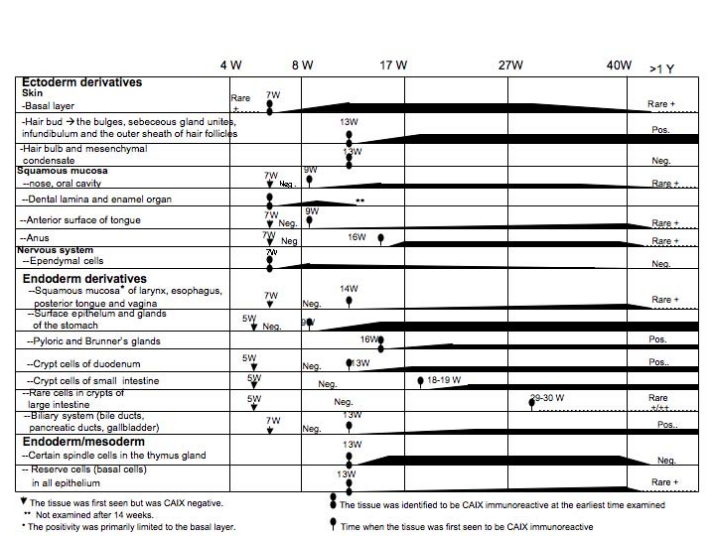
**Schema of CAIX expression in the ectodermal and endodermal derivatives during human development (embryonic, fetal and postnatal periods)**.

During the postnatal period (especially after one year of age) and throughout adult life [7], CAIX expression was no longer present in the normal tissues, with the following exceptions: 1) persistent CAIX expression in mesothelial cells of the body cavity, the surface flat epithelium of all visceral organs, the rete ovarii, rete testis, tubuli reti, the hydatids of Morgagni, the appendix of the testis and efferent ductules. With the exception of efferent ductules, all of these CAIX positive organ systems are derived from coelomic epithelium; 2) persistent high levels of CAIX expression in the GI system, comprising the gastric surface epithelium, gastric glands, pyloric/Brunner's glands, crypt cells of the small intestine, and the biliary trees, including the gallbladder; 3) high levels of CAIX expression in the infundibulum and the outer sheath of hair follicles, and the sebaceous units of the skin; 4) variable degrees of CAIX expression in the primitive reserve cells or immature metaplastic squamous cells of the lining epithelium of all visceral organs and rare columnar cells in the crypts of the large intestine. Interestingly, in the reparative squamous or respiratory mucosa, not only do the numbers of basal/reserve cells expressing CAIX appear to increase, CAIX expression is also observed in rare columnar cells; and 5) the only mesenchymal tissues that retained CAIX expression after birth were the submesothelial stromal cells, the meniscus and the nucleus pulposus of the vertebrae. Schematic representations of the temporal and tissue-specific distribution of CAIX expressing cells, both pre- and postnatal, are shown in Figs 5 and 6. The patterns of expression clearly indicate that, during human development, all of the CAIX positive cells were derived from the mesoderm and were particularly related to the embryonic coelom and mesenchyme, with the exception of the skin, squamous mucosa, the upper gastrointestinal tract and the efferent ductules.

### CAXII expression during human development

**Figure 7:**
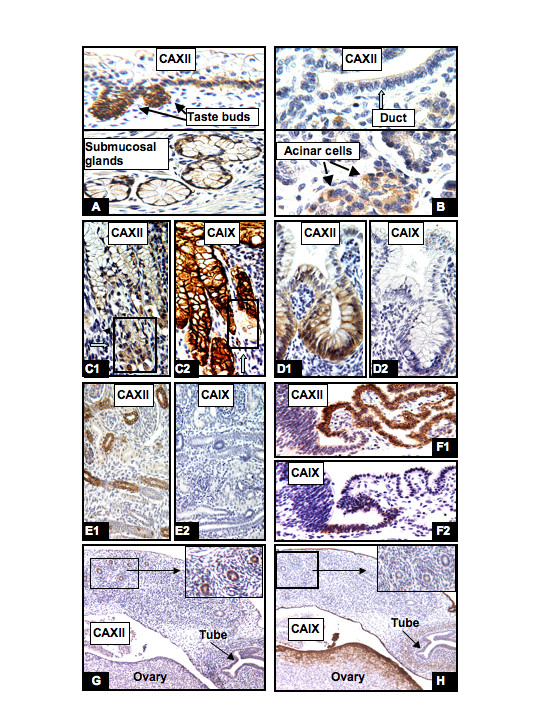
**Comparison of CAIX and CAXII expression during human development**. There is no correlation between CAXII and CAIX expression. CAXII positive cells were distributed in taste buds and the submucosal gland of the tongue (A, 17W), the acinar cells of the pancreas (B, lower panel, but not ductal cells [B, upper panel]), the parietal cells but not columnar cells of the stomach (C1, 27W), the large intestine (D1, 17W), the distal tubules and collecting ducts of the kidney (E1, 8W), the choroid plexus (F1, 8W) and the remnants of mesonephric ducts of the ovary (G, 13W). In contrast, CAIX positive cells were seen in the gastric columnar cells (C2, 27W), the ependymal cells (F2, 8W), and the surface epithelial cells of the ovary and peritoneum (H, 13W). However, CAIX expression was not seen in the large intestine (D2, 17W), kidney (E2, 8W) and the remnants of the ovarian mesonephric ducts (H, 13W). W = gestational age in weeks. Original magnifications: A, F1 and F2 (20×); B, C1, C2, D1 and D2 (40×); E1, E2, G and H (10×).

In contrast to CAIX, the expression of CAXII was limited to very few organ systems. The numbers of cells stained and the intensity of positive staining were consistent throughout the embryonic and fetal periods and into the entire adult life. Its expression was observed predominantly in cells derived from the mesonephric duct or those cells whose differentiated function involves secretion and proton pumping. The coelomic cell origin, cell differentiation and degree of cellular hypoxia (the latter playing a significant role in regulation of CAIX expression), probably play no significant role in regulation of CAXII expression. CAXII expression was first observed in syncytiotrophoblasts during the embryonic period. Between 7–12 weeks of gestation, high levels of CAXII expression were already seen in those tissues involved in secretion or pH regulation. For example, the taste buds and underlying glands of the tongue (Fig. 7A), pancreatic acinar cells (Fig. 7B) and acinar cells of the minor/major salivary glands, the parietal cells of the stomach (Fig. 7C1), the epithelium of the large intestine (Fig. 7D1), renal tubules (Fig. 7E1), and choroid plexus (Fig. 7F1). CAXII expression was also observed in the remnants of mesonephric ducts of the testis and the ovary (Fig. 7G), and the epithelium of the inner ear (data not shown). Variable degrees of CAXII immunopositivity were also observed in the basal cells of the respiratory mucosa and the squamous mucosa lining the oral cavity, esophagus, cervix, vagina and the anus (data not shown). All of the tissues that expressed CAXII during fetal development retained their expression after birth and throughout adult life [7]. A representative comparison of expression of CAIX and CAXII proteins is shown in figs 7C–7H. It appears that the majority of organ systems show no co-expression of CAXII and CAIX.

### Correlation between CAIX and HIF-1α expression during human development

**Figure 8:**
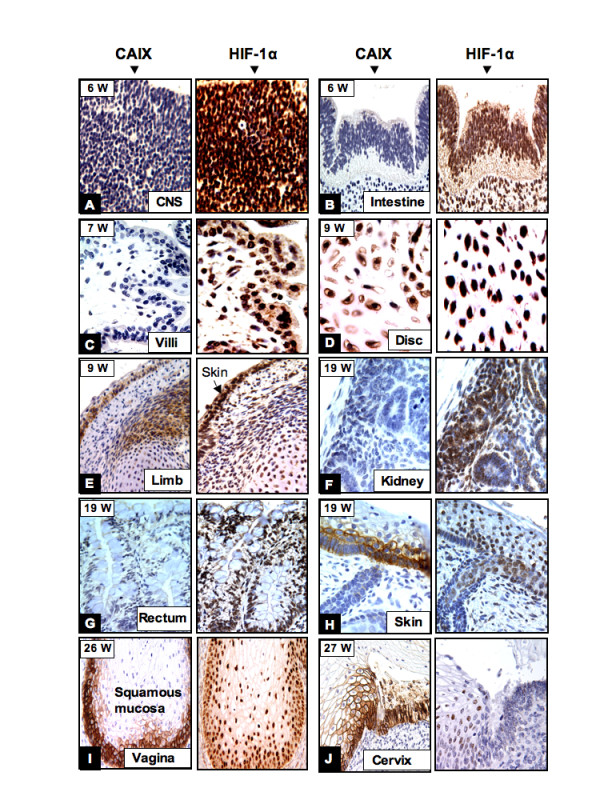
**The correlation between CAIX and HIF-1α expression during human development**. In the early stage (5–9 weeks of gestation) diffuse HIF-1α immunoreactivity was seen in the CNS (A), primitive intestine (B), chorionic villi (C) and chondrocytes of the disc (D), mesenchymal cells and chondrocytes of the limb (E), and the skin (E arrow). In contrast, no CAIX expression was seen in the CNS (A), primitive intestine (B), and chorionic villi (C). Co-expression of HIF-1α and CAIX was seen in the skin (E) and the cells involved in chondrogenesis (D, E). During later human development, around 19–20 weeks, HIF-1α expression was persistently observed in the kidney (F) and rectum (G) but no CAIX expressing cells were seen in these organs. However, a degree of overlap between CAIX and HIF-1α expression was seen in the skin (H), squamous mucosa of the vagina and the cervix (I and J). W = gestational age in weeks. Original magnifications: A, B, D-H (40×); C (left panel 20×; right panel 40×); I (20×); J (left panel 20×; right panel 40×).

Recent evidence indicates that the fetus exists in a relatively hypoxic environment. The fetal PO2 is 35 mm of Hg compared with 100 mm of Hg in the adult [23]. HIF-1α mRNA is constitutively expressed in all organs and at all stages of gestation from 14–22 weeks, with the highest expression noted in the brain, heart, kidney, lung and liver [23]. However, HIF-1α protein is rapidly degraded in the proteasome, via VHL E3 ligase recognition, under normoxic conditions and is stabilized under hypoxic conditions [2,3]. In the transgenic mouse model study conducted by Provot et al., they found that HIF-1α protein is stably expressed and transcriptionally active in limb bud mesenchyme and in mesenchyme condensations during fetal development [24]. Thus, HIF-1 appears to play an important role in early chondrogenesis and joint formation. We performed an immunohistochemical study for HIF-1α expression on the fetal tissues, representing all stages of human development from 5–38 weeks of gestation, and encompassing both embryonic and fetal development. We found that as early as 5–9 weeks of gestation, diffuse HIF-1α nuclear immunoreactivity was seen in most of the embryonic and fetal tissues, particularly in the endothelial cells, body mesenchymal cells and nervous system (Fig. 8A), primitive gastrointestinal tract (Fig. 8B), and the chondrocytes and mesenchyme of vertebral disc/limb bud (Fig. 8D, 8E). In the skin, HIF-1α expression was consistently observed during fetal development (Fig. 8E, 8H). Interestingly, the immature chorionic villi during the 1st trimester also showed diffuse HIF-1α nuclear staining (Fig. 8C) and this observation is consistent with that previously published [26]. Variable degrees of HIF-1α expression were also observed in the visceral organs, especially during 17–25 weeks of gestation. The best examples are shown in fig 8F (kidney) and 8G (rectum). Although HIF-1α expression was persistent throughout the fetal period, the numbers of expressing cells progressively diminished. During the last trimester of fetal development (27–40 weeks) stable HIF-1α expression was limited to chondrocytes, endothelial cells, skin, and epithelial cells of the squamous mucosa, such as the vagina and cervix (Fig. 8I, 8J), the oral cavity, esophagus and anus.

It has been established that CAIX is an endogenous cellular hypoxia marker in solid tumor tissues, and that HIF-1α is an important factor in the regulation of CAIX expression [2-8]. This correspondence between HIF-1α and CAIX expression was also seen in this study in certain cell types during the embryonic period to early stage of fetal development, and between 17–25 weeks of gestational age. During the embryonic and early fetal periods, those cells comprising the primitive mesenchyme, or involving chondrogenesis and skin development, expressed high levels of CAIX. These CAIX positive primitive cells either co-expressed HIF-1α or were located adjacent to HIF-1α expressing cells. The best examples were the embryonic mesenchyme and chondrocytes of the vertebrae, limb buds, and basal cells of the skin (Fig. 8D, 8E, 8H). In the 17–28 week gestation period, there were transient high levels of CAIX expression in the epithelial cells derived from the coelom and the basal layer of the squamous mucosa. Some, but not all, of those CAIX-positive epithelial cells also showed nuclear immunostaining for HIF-1α; for example, vaginal squamous mucosa and the cervix (Fig. 8I, 8J). The findings appear to indicate that CAIX expression in the developing fetal tissues is partially under the influence of hypoxia. However, it is important to note that not all of the cells expressing CAIX protein were necessarily related to the hypoxic condition and, conversely, not all of the hypoxic cells (as indicated by stable HIF-1α expression) expressed CAIX. For example, the trophoblasts of the chorionic villi were hypoxic and showed diffuse nuclear immunoreactivity for HIF-1α, as shown by us and others, but were negative for CAIX expression (Fig. 8C).

The embryonic and fetal tissues during 5–12 weeks of gestation were uniformly hypoxic. By immunohistochemical study, high levels of HIF-1α expression was seen in most of the fetal tissues but no CAIX expression was present in the majority of visceral organs; for example, the primitive nervous system (Fig. 8A), kidney (Fig. 8F), gastrointestinal tract (Fig. 8B, 8G), lungs, cardiac muscle, and liver (data not shown). In contrast, during late fetal development there were epithelial cells of coelomic derivatives, stomach, biliary system, crypt cells of the intestine and squamous mucosa of the cervix, vagina that exhibited high levels of CAIX expression; however, no HIF-1α nuclear staining was seen in those CAIX positive tissues, with the exception of squamous mucosa of the cervix and vagina, as noted above.

It has been shown that CAIX expression in the gastrointestinal tract is not only involved in maintenance of tissue integrity and regulation of basolateral ion transport but is also closely related to cell proliferation and differentiation [27]. Thus, we also performed Ki67 immunostaining, a marker of cellular proliferation, in selected cases to determine whether the postulated coordination of proliferative activity and CAIX expression is seen. In this study, we found that the majority of CAIX positive cells were not in the proliferative stage (data not shown).

## Conclusion

Although CAIX and CAXII expression during mouse embryonic development has been previously described [28], a systematic comparative study of CAIX, CAXII and HIF-1α expression in the embryonic, fetal and postnatal tissues during human development has not been reported. We find that the distributions of CAIX and CAXII immunoreactivity in the mouse tissues are similar but not identical to the immunoreactive patterns observed in the human organs. For example, staining for CAIX in the mouse embryos showed a relatively wide distribution pattern with moderate signals in the brain, lung, pancreas and liver, and weak expression in the kidney and stomach [28]. In human fetal tissues, CAIX expression in those organ systems was restricted to certain cell types, such as the primitive ependymal cells of the brain, immature mesenchymal cells of the bronchial tree system, and epithelial cells of the pancreatic ducts and stomach. High levels of CAXII expression were seen in the choroid plexus in both mouse and human tissues, but CAXII expression in the embryonic mouse kidneys and other organs was weak and became negative in the adult mice. In contrast, the intensity of CAXII expression in the human fetal tissues persists postnatally throughout the adult lifespan.

Several intriguing features concerning CAIX expression have emerged from the study of the developing human embryo and fetus. As expected, HIF-1α and CAIX expression colocalized in many, but not all, of the embryonic and early fetal tissues. As fetal development progressed, and vascularization increased, the lack of co-expression of HIF-1α and CAIX became more apparent in multiple tissues. In those instances where HIF-1α is not expressed but CAIX is, one must assume that its expression is regulated by other transcriptional factors. We, and others [16,29], have shown that SP1 is required.

Furthermore, transcription mediated indirectly by P13-kinase activity may also be a possibility [18]. Of particular interest is the situation where HIF-1α is stably expressed but no CAIX expression is found. Stable inactive HIF-1α expression has been noted in the presence of proteasome inhibitors [30]. One possibility is the expression of a co-repressor inhibiting the activity of HIF-1. Another important factor in the regulation of CAIX expression is the cell origin. CAIX expression was consistently observed in cells derived from the coelomic epithelium. The best examples are mesothelial cells, modified mesothelial cells (Müllerian epithelium), underlying mesenchymal cells lining the body cavity, and coelomic remnants, such as the rete ovarii, rete testis and tubuli reti, the hydatid of Morgagni and the appendix of the testis. Another feature of the regulation of CAIX expression is its pH control function. CAIX is a transmembrane carbonic anhydrase that possesses cell surface enzyme activity that catalyzes the conversion of CO2 into bicarbonate and protons [31]. Thus, the CAIX expression observed in the gastrointestinal tract is more likely induced by cellular acidity and the requirement for proton transport.

Although CAXII expression was originally thought to be regulated by HIF-1 [10], this is clearly not the case. There is no obvious co-localization of HIF-1α and CAXII.

Furthermore, there is also no co-localization of CAIX and CAXII. The distribution of CAXII expression would suggest that it plays an important physiological role in secretory/absorptive cells in different organ systems, primarily involving ion transport and fluid concentration. Corroborating this suggestion is a previous study that found that CAXII is overexpressed in the ciliary epithelial cells of glaucomatous eyes and may be involved in aqueous humour production [32].

A particularly intriguing finding was the discrete distribution of CAIX expression in rare cells or niches in late stages of fetal development, and postnatally, that correspond to sites previously identified as harboring adult stem cells. For example, CAIX expression in the skin is restricted to the hair follicles, including the bulge, sebaceous gland, outer root sheath and infundibulum, plus rare cells in the inter-follicular zone. This corresponds to epidermal stem cell niches that have been described, using various stem cell biomarkers [33,34]. Also, the distribution of CAIX expression in the small and large intestine is very similar to that described for the identification of the intestinal stem cell niche, using the Lgr5 biomarker [35]. Finally, our observation of CAIX expression in Müllerian-type columnar cells and reserve cells of the cervix, during fetal development and postnatally, appears to be similar to that described in the study conducted by Martens and colleagues [36]. These authors claim that the Müllerian epithelial cells represent the stem cells for endocervical reserve cells and columnar cells. Thus, we hypothesize that the rare reserve cells expressing CAIX in the cervix and other organ systems may correspond to putative stem cell regions. However, at this time, such distributions and their relevance to stem cell identity are speculative and will require more comparison and functional analyses. These studies are in progress. If CAIX is found to be a stem cell marker for certain tissues it will be an attractive one. Its transmembrane location will be extremely useful for cell sorting analyses. It may also provide a caveat for cancer therapeutic regimens that target CAIX-expressing cells for destruction [37].

## Methods

### Tissue specimens

The embryonic, fetal, and postnatal tissues were collected at St. Joseph Hospital (Orange, CA) from 1997 to 2005. The sources of these specimens included spontaneously abortive embryos, stillborn fetuses and organs of autopsy cases. Consent was obtained prior to any medical procedures being performed. The extra-tissue sections were prepared and labeled precisely according to the age distribution without any identification. The studied cases included 10 from the embryonic period (4 to 8 weeks), 42 from the fetal period (9 weeks to birth) and 26 from the postnatal period (one day to 8 years old). The cases from the fetal period were further divided into 9 to 12 weeks (5 cases), 13 to 16 weeks (9 cases), 17 to 20 weeks (7 cases), 21 to 25 weeks (4 cases), 26 to 40 weeks (17 cases). All of the tissues were fixed in 10% neutral-buffered formalin, paraffin-embedded, sectioned, and stained with hematoxylin and eosin (H&E).

### Immunohistochemical studies

The mouse monoclonal antibody (M75) used to detected the MN/CAIX protein and the rabbit polyclonal antibody to CAXII have been described previously and both antibodies have been shown to be specific for CAIX and CAXII, respectively [38,39].

Immunohistochemical staining of tissue sections with anti-CAIX and anti-CAXII antibodies was done using a peroxidase technique with a pressure cooking pretreatment.

The peroxidase method and the primary antibody dilution for CAIX (1:10,000) and CAXII (1:500) have been described previously [7,12,14]. For CAXII immunostaining, the avidin-biotin enzyme complex Kit (LSAB2, DAKO Corp., Carpinteria, CA) was used. Positive and negative controls were included in each run. Anti-HIF-1α (NeoMarkers INC. Fremont, CA, USA) and anti-Ki67 (Dako) mouse monoclonal antibodies at a 1:100 dilution, respectively, were also applied to selected cases. For HIF-1α, the sections were exposed to pressure cooking pretreatment in citrate buffer (pH 6.0) for 15 min and the DAKO Catalyzed Signal Amplification System (CSA) was used according to the manufacturer's instructions.

## Abbreviations

CAIX: carbonic anhydrase IX; CAXII: carbonic anhydrase XII; HIF: hypoxia inducible factor.

## Authors' contributions

SYL and EJS conceived of the study and participated in its execution. SYL, EJS and MIL participated in its design and drafting of the manuscript. All authors read and approved the final manuscript.
